# The “TASK” of Breathing: Anesthetic Relevance of Background Two-Pore Domain Potassium Channels as Therapeutic Targets for Respiratory Control

**DOI:** 10.1213/ANE.0000000000007365

**Published:** 2025-02-13

**Authors:** Ann Y. Lin, Christopher D. Turnbull, Jaideep J. Pandit

**Affiliations:** From the 1 Nuffield Department of Anaesthetics, Oxford University Hospitals NHS Foundation Trust, Oxford, United Kingdom; 2 Department of Respiratory Medicine, Oxford University Hospitals NHS Foundation Trust, Oxford, United Kingdom; 3 Nuffield Department of Medicine, University of Oxford, Oxford, United Kingdom; 4 Nuffield Department of Clinical Neuroscience, University of Oxford, Oxford, United Kingdom.

## Abstract

Background (leak) potassium (K+) currents, the main contributors to resting membrane potential in excitable cells, are mediated by channels of the 2-pore domain (K2P) family. In the respiratory system, the TWIK-related acid-sensitive K+ channel (TASK) subfamily is proposed to mediate key functions in the carotid body type I glomus cells, central chemoreceptors and respiratory center, pulmonary arteries, and upper airway musculature. K2P channels are also located throughout the central nervous system, notably in the hypoglossal motor neurone pool, regions involved in sleep-wake regulation and pain perception. Being sensitive to general anesthetics, K2P channels may mediate both the adverse respiratory effects and hypnotic actions of many anesthetics. Therefore, they offer potential as pharmacological targets to reverse postoperative respiratory depression, ameliorate anesthetic risks of obstructive sleep apnea, improve ventilation-perfusion matching, and even assist in the active recovery from hypnotic effects of anesthesia during emergence from surgery.

Potassium (K+) channels of the 2-pore domain (K2P) family provide background conductances whose regulation determines cell membrane excitability.^1^ Structurally and functionally they are unlike voltage-gated (Kv) or inward-rectifying (Kir) channels (Figure 1).^2–5^ K2P channels permit passive “leak” of K+ from within the cell, thus helping maintain the negative membrane potential. The K2P family has been classified into 6 subfamilies with varying properties and expression patterns: TWIK (2 P-domain in weakly inward-rectifying K+ channel); TREK (TWIK-related K+ channel); TASK (TWIK-related acid-sensitive K+ channel); TALK (TWIK-related alkaline-sensitive K+ channel); THIK (tandem pore domain halothane-inhibited K+ channel); and TRESK (TWIK-related spinal cord K+ channel)^1^ (see Supplemental Digital Content, Online Supplement S1, http://links.lww.com/AA/F214 for the phylogenetic relationships between these).

**Figure 1.**
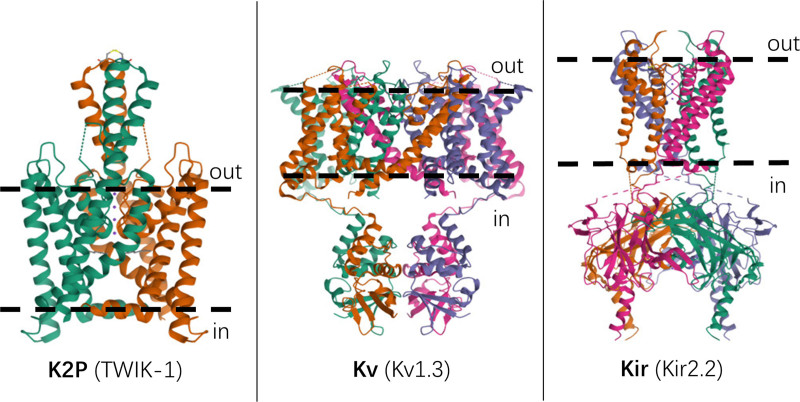
Structures of representative 2-pore domain (K2P), inward-rectifying (Kir) and voltage-gated (Kv) K+ channels, taken from the Research Collaboratory for Structural Bioinformatics Protein Data Bank.^2^ Dashed lines indicate approximate positions of plasma membranes, which were derived from the experimental sources for these structures: X-ray crystallography data for TWIK-1 and Kir2.2 by Miller and Long^3^ and Tao et al^4^ respectively, and cryo-electron microscopy data for Kv1.3 by Selvakumar et al.^5^ Not to scale.

The widespread importance of K2P channels is increasingly evident in the respiratory^6^ and central nervous systems (CNS; where they are likely mediators of narcosis and analgesia).^7^ Postoperative respiratory depression is a common phenomenon arising synergistically from use of general anesthetics with neuromuscular blockers and sometimes opioids^8^; Hao et al^9^ provided an overview of how anesthetics and opioids modulate the respiratory system. We will complement their analysis and outline the tissues of the body involved in respiratory control where K2P channels are distributed (with a focus on TASK), and summarize the known influence of anesthetics on channel and tissue function (Figure 2). We will also discuss how targeted inhibition of anesthetic action at this receptor site could help prevent the adverse effects of anesthetics, along with other potential therapeutic benefits of modulating K2P activity.

**Figure 2.**
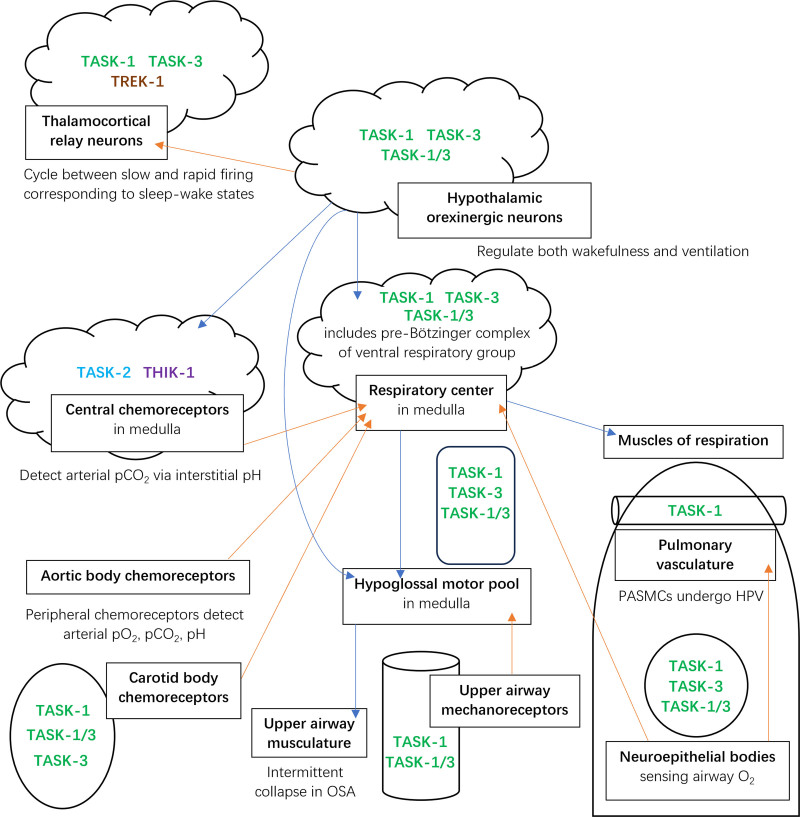
Schematic of components of respiratory and central nervous systems with their interconnections. Arrows indicate feedback or feedforward signaling by direct or indirect means. K2P channels are listed at sites where this paper has discussed their roles. In the hypoxic ventilatory response (HVR), sensing of hypoxia by carotid body chemoreceptors leads to excitation of glossopharyngeal nerve terminals and signaling to the respiratory center, which in turn stimulates hyperventilation. Pulmonary artery smooth muscle cells (PASMCs) undergo hypoxic pulmonary vasoconstriction (HPV), as discussed in the article.

## K2P CHANNELS MEDIATE SENSING OF HYPOXIA AT CAROTID BODY GLOMUS CELL

At clinically relevant concentrations, volatile anesthetics open K2P channels.^10–13^ There is some variation in response: the anesthetic gas N_2_O activates TREK-1 and TRESK but not TASK-3^11,12^; similarly, xenon and cyclopropane activate TREK-1 but not TASK-3^11^; chloroform inhibits TASK-1,^10,13^ and halothane inhibits THIK-1^14^ (Supplemental Digital Content, Online Supplement S2, http://links.lww.com/AA/F214, summarizes work in this area). Wague et al^15^ identified in TREK-1 a volatile anesthetic binding site that overlaps with positions known to affect TASK channels’ anesthetic sensitivity.

**Figure 3.**
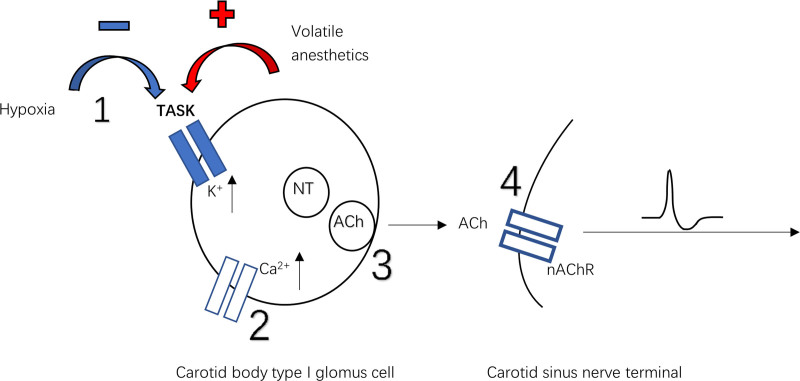
Schematic of oxygen sensing at carotid body glomus cell, based on Raju and Pandit.^20^ Hypoxia inhibits TASK channels (1), leading to intracellular K^+^ accumulation and depolarization. Depolarization activates voltage-gated Ca^2+^ channels (2), and Ca^2+^ enters the cell. This leads to fusion of neurotransmitter (NT)-containing vesicles with the synaptic membrane (3). Released acetylcholine (ACh) binds to nicotinic receptors (nAChR) postsynaptically (4), triggering action potentials.

The carotid body has long been established to mediate the hypoxic ventilatory response (HVR chemoreflex). Buckler et al first described a TASK-like background K+ current inhibited by hypoxia and acidosis and activated by halothane in rat glomus cells,^16^ later confirmed to be mediated mostly by TASK-1/3 heteromers, along with TASK-1 and TASK-3 homodimers.^17,18^ Thus single- and double-TASK knockout mice show reduced responses to hypoxia in terms of glomus cell Ca^2+^ signaling, carotid sinus nerve discharge, and ventilation.^18,19^ Figure 3 shows the sequence of events within the carotid body: hypoxia-induced closure of TASK channels causes membrane depolarization, voltage-dependent Ca^2+^ channels open, and this results in vesicle fusion with the membrane then neurotransmitter release to act on the postsynaptic terminal of the carotid sinus nerve to trigger the HVR chemoreflex. Key questions remain as to exactly how oxygen is sensed (eg, whether the TASK channel is directly sensitive to O_2_ or regulated more indirectly by O_2_ levels).^21^

Before the work of Knill’s group in the 1970s, it was erroneously believed that this HVR chemoreflex was resistant to anesthesia, which would be reassuring for patient care. However, in an elegant series of human volunteer and clinical studies, Knill and others showed that even at very low residual concentrations (<0.2 MAC), volatile anesthetics greatly depress the acute hypoxic ventilatory response (AHVR).^22,23^ These are concentrations that persist long after surgery and plausibly contribute to postoperative ventilatory depression, which is well established to be an important factor in postoperative morbidity and mortality—the effects being worse in patients with preexisting cardiorespiratory disorders.

Yet, even as late as the 1990s, some authoritative reviewers were rejecting the possibility that direct inhibition of the carotid body’s glomus cell was responsible for this anesthetic effect.^24^ However, the sheer weight of evidence overturned this view, and experiments in isolated animal glomus cells confirmed the direct depressive effects of agents.^25,26^ Moreover, the rank order of potency of anesthetics in depressing the HVR in humans was shown identical to that in animals and the isolated glomus cells.^23^ Finally, single channel (patch clamp) recordings in native (rat) TASK channels confirmed their activation by halothane, isoflurane, and sevoflurane^25,26^ again with the order of potency of effect mimicking the rank order observed in human studies. The fact that single channel recordings of TASK channels expressed in human embryonic kidney (HEK) cells were also influenced by anesthetics suggests a direct effect on channels,^27^ since HEK cells are “passive” entities devoid of cellular machinery to regulate TASK-channel activity, that could confound interpretation of results. Therefore, these studies confirmed with little doubt that the volatile anesthetic effect was a direct one on the channel itself. In this way, the opening of TASK channels by volatile agents counteracts their closure by hypoxia, explaining the HVR depression seen in human volunteers and patients.

In contrast to these actions of volatile agents, many intravenous anesthetics (etomidate, pentobarbital, ketamine, and alphaxolone) inhibit many K2P channels, albeit at supraclinical concentrations^12,28,29^ (Supplemental Digital Content, Online Supplement S2 for the intravenous data, http://links.lww.com/AA/F214). If these actions occur at the same molecular binding site as that for the volatile agents, this raises the possibility of competitive, mutually antagonistic interactions between anesthetics; that is, the depressive effect of a volatile agent on HVR could be prevented, mitigated, or reversed if coapplied with a suitable intravenous agent. While such mixtures have not yet been tested experimentally, Pandit et al coapplied mixtures of halothane and isoflurane to isolated glomus cells (measuring hypoxia-evoked Ca^2+^ influx and native TASK-channel activity), and to HEK cells expressing TASK-1 subunits.^30^ Halothane behaved as a “full agonist,” completely inhibiting hypoxia-evoked Ca^2+^ responses in glomus cells and maximally activating native and expressed TASK channels; while isoflurane behaved as a partial agonist, with only a weak effect even at supraclinical concentrations. As would be predicted by classical pharmacology, in mixtures of these agents isoflurane antagonized the effects of halothane in a manner quantitatively predicted by competition at a single binding site. Such mutually antagonistic, competitive interactions between agents have also been described at the GABA receptor by Forman^31^ for the intravenous agent etomidate; and suggested for interaction between N_2_O and sevoflurane on EEG parameters in patients.^32,33^ Both these examples do not involve K2P channels but illustrate the principle that anesthetic agents can compete with each other infraadditively at clinically relevant single molecular targets.

Propofol has no effect on TASK channels,^28,34^ but it does depress glomus cell response to hypoxia, in line with depression of HVR.^34^ Its mechanism appears to be non-TASK, non-GABA, nonserotonin, and noncholinergic; possibly via an action on voltage-gated Ca^2+^ channels. This underlines the message that agents can depress the glomus cell and HVR by non-TASK mechanisms.

There are 2 relevant implications of these exciting results of mutual antagonism between anesthetics. One is that, on a scientific level, it challenges the Meyer-Overton theory that anesthetics primarily act via the nonspecific mechanism of dissolution in the lipid membrane. This was already challenged long ago by the finding of Ueda, who first demonstrated that 2 structurally dissimilar volatile anesthetics (diethyl ether and halothane) inhibited firefly luciferin bioluminescence^35^; a finding extended by Franks and Lieb.^36,37^ On a practical level, this opens the door to design specific antagonists to the undesirable HVR-depressant effect, whilst preserving the beneficial hypnotic actions. Doxapram is now confirmed to act in this way at TASK channels,^38^ and helps restore breathing postoperatively when used judiciously.^39^ Although doxapram is explicitly approved by the US Food and Drug Administration (FDA) “to stimulate respiration in patients with drug-induced postanesthesia respiratory depression or apnea other than that due to muscle relaxant drugs,”^40^ it is contraindicated in patients with seizure disorders or head injury, severe coronary artery disease, and uncompensated heart failure. This highlights the role K2P channels play in other pathologic conditions, as documented elsewhere.^1,41^ Drug development in this space is difficult and slow.^42^ Other experimental agents with greater specificity for the TASK channel include PK-THPP, A1899, and ML365.^27,43^

## ROLES OF TASK AND OTHER BACKGROUND K+ CHANNELS IN CENTRAL RESPIRATORY MECHANISMS

Given their pH-sensitivity, TASK channels have naturally also generated interest as mediators of central chemoreception.^44^ TASK-1 and TASK-3 in homo- and heterodimer forms are widely expressed in the CNS, including brainstem chemoreceptor populations.^44,45^ TASK-2, which belongs to a different K2P subgroup than TASK-1 and TASK-3 (Supplemental Digital Content, Online Supplement S1, http://links.lww.com/AA/F214), has a restricted distribution in the CNS, limited to some discrete areas in the brainstem.^46^ TASK-1 and TASK-3 homo- and heterodimers have pH–conductance curves distinct from those of TASK-2 homodimers; however, they all fundamentally show substantial increases in conductance with alkalization in the physiological pH range.^45^

There is robust in vitro, in situ and in vivo evidence indicating that TASK-2 is involved in central chemosensitivity.^45^ Within the medulla, TASK-2 colocalizes exclusively with the Phox2b-expressing respiratory chemoreceptor neurons of the retrotrapezoid nucleus (RTN).^47^ In vivo studies have shown that activation or inhibition of these neurons modulates ventilation.^47^ Mutations of the homeobox gene PHOX2B cause central congenital hypoventilation syndrome (CCHS).^48,49^ The defining characteristic of CCHS is a disabling central sleep apnea, although the syndrome also includes a diffuse pathology of the autonomic nervous system.^50^ Daytime breathing is modestly impaired and is still activated normally by arousal, cognitive activity, or exercise^51,52^ but respiratory stimulation by hypoxia and hypercapnia is severely attenuated during both waking and sleep.^50^

Wang et al found using patch-clamp recordings that while control RTN neurons were nearly all pH-sensitive, only 56% of RTN neurons from TASK-2 KO mice were pH-sensitive,^47^ thus estimating the degree to which TASK-related mechanisms might contribute to central chemoreception. Moreover, in a working heart-brainstem preparation, TASK-2 KO blunted inhibition of phrenic nerve burst amplitude during respiratory alkalosis and increased the pH threshold for apnea.^47^ In their TASK-2 KO mouse model, Gestreau et al reported that TASK-2 stabilizes the membrane potential of central chemoreceptive cells.^46^ They found that hypoxia-induced respiratory depression was abolished in TASK-2 KO mice, and hypothesized that TASK-2 activation by reactive oxygen species which are generated in hypoxia could silence the RTN neurons, as a basis for this respiratory depression.

Evidence appears equivocal on whether TASK-1 and TASK-3 are also important in central chemoreception. Whole-body plemysthography in mice knocked out for TASK-1, TASK-3 or both showed no differences in increases in ventilation under hyperoxic hypercapnia (hyperoxia was used to isolate the central chemoreceptor effect); also, in RTN neurons, knockout did not change pH-dependent firing or pH-sensitive background K+ current.^44^ Conversely, more recently in a rat model, injecting anandamide or ruthenium red into the ventrolateral medulla increased phrenic nerve discharge and shortened inspiratory time.^53^ These agents were respectively thought to be specific antagonists for TASK-1^54^ and TASK-3.^21^ However anandamide has since been shown to inhibit both TASK-3 and the TASK-1/3 concatamer.^21^ Ruthenium red also inhibits Ca^2+^ channels, but in the context of K2P channels is reasonably selective.^55^

Another K2P channel implicated at central chemoreceptors is THIK-1 (TWIK-related halothane-inhibited). In contrast to halothane’s effect on this channel, isoflurane was reported to activate RTN chemoreceptor neurons via inhibition of a THIK-1-like current,^56^ raising the possibility that—as with TASK in the carotid body—agents may work in mutually antagonistic ways.

The observation that some K2P subtypes are activated by anesthetics and others inhibited raises questions as to how the whole-body response (depression of AHR by anesthetics) then arises. One possibility is that there is differential expression of the channels across tissues (and varying by species) such that the final AHR whole-body response to a given agent represents the balance of activated and inhibited receptors. However, somewhat against this possibility, Rajan et al have shown the inhibited THIK channels are strongly and ubiquitously expressed across the brain.^14^ Another possibility is differential potency of agents (ie, that those agents having activating effects on targets have higher potency than the inhibitory effect on other channels, such that the whole-body respiratory response is one of depression). Coupled with this is that the experimentally measured potency at a given receptor is likely dependent greatly on experimental conditions; ie, the studies examining TASK (activated by anesthetic) and THIK (inhibited) are undertaken by different groups using different experimental models. This includes a mix of in vivo settings using, for example, native channels such as within glomus cells of carotid body, and in vitro conditions such as in expressed channels. Direct comparison of potency across all these channels has not, to our knowledge, been undertaken. Also relevant is the discovery of endogenous mechanisms by which these channels are silenced by a process known as “SUMOylation.” It has been reported that TWIK-1, present at the plasma membrane, is silenced by binding of a small ubiquitin-like modifier (SUMO) polypeptide.^57^ TWIK-1 can heteromerize with TASK-1/3 subunits and brings SUMOylation sensitivity to the TWIK-1/TASK heteromeric complexes.^58^ In other words, SUMOylation/deSUMOylation could be a dynamic way in which K2P channels might be endogenously regulated, including in their sensitivity to anesthetics.

Another question warrants even more speculation and is the “evolutionary” one as to why there have evolved K2P channels which have diametrically opposed responses to anesthetics. One speculation is as follows. Where there is an endogenous ligand, it is generally advantageous for there to be a feedback inhibitory mechanism to facilitate control. An example is that of catecholamines binding to presynaptic α2 catecholaminergic receptors, which serves to limit postsynaptic excitatory actions on α1 receptors. In context of K2P channels, we do not know which endogenous ligand this could be, but the principle that something like this might explain the evolution of anesthesia has been suggested by Sonner.^59^

The respiratory center, responsible for respiratory rhythm generation, is functionally and anatomically distinct from the central chemoreceptor region. Koizumi et al^60^ found that TASK currents contribute in the pre-Bötzinger complex (pre-BötC), an excitatory network of neurons in the medulla essential for inspiratory rhythmogenesis. They deduced that K2P channels are dominant contributors to the leak current of pre-BötC neurons, discovered that the leak conductance was reduced by acid and increased by halothane; and found that (as decreasing K+ leak conductance causes depolarization) transient application of acid increases the intrinsic bursting frequency of pre-BötC neurons. They further established by single-cell RT-PCR that the neurons expressed TASK-1 and a subset also expressed TASK-3 mRNA.

Therefore, to summarize, general anesthetics depress ventilation and ventilatory responses through mechanisms involving K2P channels, predominantly TASK, at peripheral and central chemoreceptors, and in the respiratory center. More recent results suggesting closing of some K2P currents by anesthetics, rather than opening, or partial agonist behavior, pave the way for targeted drug development—or intriguing use of combinations of agents in clinical practice—to prevent or mitigate any respiratory depressive effects.

## TASK CHANNEL MEDIATION OF HYPOXIC PULMONARY VASOCONSTRICTION (HPV)

The vasoconstriction of pulmonary arterioles to local hypoxia (HPV) achieves ventilation-perfusion matching for efficient gas exchange, and TASK channels have been found to underlie the response. After initial identification of a low-threshold, noninactivating hypoxia-sensitive potassium current I_KN_ through whole-cell patch-clamp experiments in rabbit pulmonary artery myocytes,^61^ Gurney et al^62^ provided strong evidence that I_KN_ was mediated by TASK-1. TASK-1 mRNA and protein were present, and I_KN_ had the “diagnostic” TASK-1 pharmacological properties of enhancement by halothane, inhibition by Zn^2+^ and anandamide, and insensitivity to cytoplasmic Ca^2+^, among other corresponding features. Confirming TASK-1’s functional importance, TASK-1 targeted RNA interference in human pulmonary artery smooth muscle cells (PASMCs) depolarized the resting membrane potential and abolished sensitivity to hypoxia, acidosis, alkalosis, anandamide, and treprostinil.^63^ Of note, TASK-1 KO mice have been shown in 2 studies not to be different from WT in their hypoxia-invoked rise in pulmonary arterial pressure, indicating TASK-1 is not important for HPV in mice.^64,65^ Rat,^66^ rabbit,^61,62^ or human^63^ models should be used instead, where TASK-1 is known to be key in the HPV.^65^

Sustained HPV over wide areas of the lung results in pulmonary hypertension, and patients with idiopathic pulmonary hypertension have reduced TASK-1 mRNA expression in pulmonary arteries, lower TASK-1 protein levels in their lung tissues, and reduced TASK-1 activity in cultured PASMCs.^66^ Pathological TASK-1 allele (*KCNK3*) mutations have been identified in patients with familial or idiopathic pulmonary hypertension,^67^ including a homozygous form associated with an aggressive familial form of the disease.^68^ In a rat model, A293-mediated inhibition of TASK-1 caused pulmonary hypertension, while TASK-1 activator ONO-RS-082 reduced stimulus-evoked pulmonary vasoconstriction.^66^

These results are consistent with the well-established finding that volatile anesthetics inhibit HPV and worsen ventilation-perfusion matching^69^ while intravenous agents have less of an adverse effect,^70^ consistent with the discussion above in relation to carotid body function. Moreover, other K+ channels are less likely involved in HPV. In isolated rabbit lungs, Liu et al found that K_V_ channel inhibition with 4-aminopyridine limited, but did not abolish attenuation of HPV by isoflurane, whilst K_Ca_ channel inhibition with iberiotoxin enhanced it.^71^ K_ATP_ channel inhibition had no effect. Attenuation of HPV by sevoflurane was not impacted by any of these K+ channel inhibitors.

Neuroepithelial bodies (NEB) are innervated groups of neuroendocrine cells located diffusely in the airway epithelium.^72^ There has been much speculation on their functions and they may act as airway oxygen sensors, releasing serotonin in response to hypoxia, and thus potentially contributing to HPV either through local action on surrounding lung vasculature or via a complex chemotransduction pathway to the medullary respiratory center.^72^ In the H146 cell line model for NEB cells, the oxygen-sensitive K+ current was found to have characteristics of TASK-1 and TASK-3: pH dependence, activation by halothane, blockade by arachidonic acid, and tetraethylammonium and dithiothreitol insensitivity.^73^ Moreover, antisense knockdown of hTASK1 and hTASK3 abolished the H146 cells’ response to acute hypoxia.^73^

As with the results of K2P roles in carotid body chemoreflex response, these results raise the possibility that drug development targeted to K2P channels, and TASK in particular, may help modulate HPV. Beneficial effects on ventilation-perfusion matching would be relevant for both anesthesia practice and the treatment of pulmonary hypertension.^66^

## TASK CHANNELS AND OBSTRUCTIVE SLEEP APNEA (OSA)

OSA is a highly prevalent condition affecting almost 1 billion individuals worldwide.^74^ OSA is defined by repeated episodes of complete (apnea) or partial (hypopnea) upper airway collapse during sleep, due to reduced upper airway muscle tone, with consequent intermittent hypoxemia, hypercapnia and arousal.^75^ Patients with OSA are at higher risk of perioperative complications such as hypoxemia, difficult intubation, atelectasis and unexpected ICU admission.^76^

General risk factors or associations apart (eg. high BMI, male sex, and older age), the precise pathophysiology underlying OSA appears to vary considerably between individuals.^75,77^ Anatomical predispositions to pharyngeal narrowing include a large neck circumference, adipose tissue deposition within upper airway structures such as the tongue, tonsillar enlargement and retrognathia.^77,78^ In addition, functional contributions include low respiratory arousal threshold, high loop gain and reduced responsiveness of the genioglossus muscle. A low respiratory arousal threshold; i.e, waking up easily with airway narrowing, is found in up to 37% of patients. A similar proportion appears to have a high “loop gain” of their hypoxic chemoreflex. The hypoxia induced by airway obstruction causes an exaggerated hyperventilation, so there are extreme swings of hypo- and hyperventilation.^75,77^

Another important factor appears to be reduced responsiveness of genioglossus, the largest upper airway dilator muscle, to negative pharyngeal pressure.^75^ Normally, upper airway mechanoreceptors, sensing airway narrowing, stimulate impulses in the hypoglossal motor pool, originating in the CNS, to activate genioglossus to reverse the narrowing. Impairment of this reflex may be involved in OSA,^79^ and TASK channels may play a role.

The observation that topical administration of local anesthetics to the upper airway inhibits genioglossus EMG activity, worsening OSA in existing patients,^80,81^ led Wirth et al^82^ to consider a complementary pharmacological approach of *augmenting* neuronal activity via topical administration of K+ leak channel blockers to increase upper airway reflex activity. The crude idea was that a K+ channel blocker, by depolarizing the cell membrane, would enhance the response to negative pressure in a manner opposite to the effect of local anesthetics. In a pig model of OSA, they serendipitously used AVE0118 which blocks, amongst other channels, TASK-1.^83^ They convincingly demonstrated that AVE0118 sensitized and amplified the activation of the genioglossus muscle in response to negative pressure, and upper airway collapsibility was dose-dependently inhibited. Clinical trials inspired by these results have had mixed results. Gaisl et al^84^ intranasally administered the TASK-1 and TASK-3 blocker BAY2253651 but with no effect on the severity of OSA as measured by the apnea-hypnopnea index (AHI) in patients with moderate to severe OSA. Osman et al used the TASK-1/3 heterodimer channel antagonist BAY2586116 showing improved upper airway collapsibility in pigs and modest improvements in upper airway collapsibility in 12 patients with at least mild OSA; they did not measure the impact of BAY2586116 on the severity of OSA.^85^ In a further randomized crossover study including 10 patients from their initial study,^86^ Osman et al found no overall effect of one night of BAY2586116, with or without the use of chin-strap to isolate nasal rather than oronasal breathing, on the severity of OSA as measured by the AHI when compared to placebo. Seven of those included met *a priori* definitions as responders and had a modest reduction of 22% in the AHI.

TASK channels are also important in the function of the hypoglossal motor pool in the CNS, these nerves providing central input to upper airway dilator muscle activity. The excitability of hypoglossal motor neurons is modulated via K+ channel activation and inhibition, in turn regulated by changes in neurotransmitter signaling between awake, NREM and REM sleep stages.^7^ Noradrenergic stimulus is withdrawn going from awake into NREM sleep, reducing pharyngeal muscle contractility.^87^ In NREM sleep, K2P channels including TASK-1/3 are thought to be the main factor controlling genioglossus tone.^88^ Li et al found that chronic intermittent hypoxia, which occurs in OSA, upregulates TASK-1 expression in the hypoglossal motor nucleus, and this upregulation is reduced by a serotonin 2A receptor antagonist.^89^

However, this theory was not supported by the observation that TASK inhibition at the rat hypoglossal motor pool by direct microperfusion is ineffective at improving baseline tongue motor activity or its 5-HT responsiveness during sleep. Grace et al tested modulators of Kir, K2P and Kv channels^88^; the TASK blocker methanandamide was only successful in wakefulness, while the Kir and Kv channel blockers increased genioglossus activity during both NREM and REM sleep. Further experimentation with TASK channel inhibitors doxapram, A1899, ML365, as well as TASK activator terbinafine, all with and without coapplied 5-HT, in anesthetised, freely behaving or sleeping rats, failed to demonstrate any TASK-dependence on tongue motor activity or 5-HT responsivity.^90^

Nevertheless, TASK channels could play a role in setting the respiratory arousal threshold and “loop gain” of the respiratory control system, discussed above.^75^ The hypothalamic orexinergic neurons regulate both wakefulness and ventilation; the latter via projections to central respiratory nuclei, central chemoreceptors, and hypoglossal motor neurons.^91^ Selective loss of orexin neurons causes narcolepsy type 1, a sleep disorder causing excessive sleepiness and cataplexy,^92^ and patients with narcolepsy type 1 have reduced hypoxic ventilatory responses.^93^ Orexin neurons are pH-sensitive, and this is established to be mediated via TASK.^91^ When TASK-1 and/or TASK-3 were blocked by microinjection of antagonists into the lateral hypothalamus, TASK-3 blockade stimulated breathing while TASK-1 blockade influenced pH-sensitivity.^94^ Hence, TASK functioning at the orexinergic neurons is likely to be another level at which OSA could be modulated. Interestingly, reduced levels of orexin are seen in patients with OSA,^95^ and orexin knockout mice show exaggerated sleep apneas^96^ and an attenuated hypercapnic chemoreflex.^97^ It is also notable that orexin signaling is involved in the regulation of feeding and metabolism, and knockout mice have a tendency for obesity,^98^ a possible basis for mechanistic link between obesity and OSA.^91^

To underscore the therapeutic potential of TASK modulation, Sormann et al^99^ identified in a human genomic analysis 9 probands with de novo gain-of-function mutations in TASK-1, all of whom had sleep apnea alongside developmental abnormalities. They reported a mixture of obstructive and central sleep apnea, so it is not clear whether gain-of-function mutations in TASK-1 cause sleep apnea by acting on respiratory control sensors, on the upper airway, or both. Furthermore, in a study of 164 patients with severe OSA and 171 patients without OSA, 2 genetic variants of the TASK-1 gene were a risk factor in obese patients for developing severe OSA versus no OSA.^100^ Also, it has been reported that chronic intermittent hypoxia (CIH) enhances inhibition of carotid body TASK channels^101,102^; this may help explain the heightened carotid body responsivity to acute hypoxia seen under CIH. CIH, the cycling of hypoxia-normoxia, has been argued to contribute to cardiorespiratory changes seen in OSA patients, including systemic and pulmonary hypertension.^103,104^ Interestingly, CIH has also been found to act centrally at the inspiratory rhythm-generating pre-Bötzinger complex,^105^ where TASK has already been discussed to contribute.^60^ Electrophysiological recordings of mouse brainstem slices revealed CIH enhances burst-to-burst irregularity of pre-BötC firing and alters transmission from pre-BötC to the hypoglossal motor nucleus, in a manner which may perpetuate apneas and other forms of respiratory instability.^105^ TASK at the pre-BötC as well as the carotid body may provide a link between hypoxia and respiratory dysautonomia in OSA.

## CENTRAL NERVOUS SYSTEM K2P CHANNELS: MEDIATION OF HYPNOTIC EFFECTS AND RELEVANCE FOR RESPIRATION

Amongst others, Franks and Honoré have suggested that suppression of neuronal excitability via activation of K_2P_ channels contributes to anesthesia.^106,107^ As Franks observed, these channels are also found presynaptically, where anesthetic-induced opening could be either inhibitory or excitatory and, depending on whether the synapses were themselves excitatory or inhibitory.

Steinberg et al have reviewed the central role of K2P channels and suggested that, interacting with neurotransmitters such as noradrenaline, acetylcholine and histamine^7^ they may influence the arousal state, but not at its “source.” Closure of TASK-1/TASK-3 and TREK-1 in thalamocortical relay neurons may be critical for the neurons to switch to a tonic firing mode associated with wakefulness and REM sleep.^108^ Given that a TASK-3 channel antagonist promotes wakefulness in rodent electroencephalogram (EEG) telemetry models^109^ and TASK-1 and TASK-3 knockout attenuates anesthetic-induced hypnosis^110–113^ (Supplemental Digital Content, Online Supplement S3 for data on hypnotic effects, http://links.lww.com/AA/F214), TASK antagonism could potentially reverse anesthesia alongside its effects on ventilation. The implications for anesthetic science and practice of such putative “antagonists” to the hypnotic effect is self-evident: in theory if K2P channels play an important role, and if specific antagonists can be developed, patients could be awoken from anesthesia not just by waiting passively for excretion or metabolism of the agent(s), but by drug administration actively to reverse hypnosis. This would offer greater control in the emergence/tracheal extubation phase of anesthesia.

The relevance of a central role for K2P channels for our review is that it is well established that the prevailing level of arousal strongly affects the ventilatory response to hypoxia. Thus, HVR is augmented in exercise,^114^ in part by the “central drive” to breathe harder; and is obtunded during sleep.^115^ This arises from interaction of anesthesia and CNS arousal mechanisms within the central arc (CNS) of the chemoreflex. These interactions between arousal and HVR differ for different agents, and for different arousal stimuli.

Thus, Dahan’s group noted that the HVR depressive effects of low dose isoflurane could be reversed by audiovisual stimulation (watching television) but not by painful stimulation (supramaximal electrical impulses).^116^ However, Pandit’s group reported that the effects of a more potent depressant of HVR, halothane, are not reversed by either audiovisual stimulation or pain.^117^ Therefore, it appears that the effects of increasing arousal of whatever particular type is agent-specific. Reversing or blocking the effects of anesthetics by antagonism at K2P channels may promote arousal and recovery from hypnosis. For some agents (like isoflurane) this may, through these interactive effects with arousal, help restore the hypoxic chemoreflex. For other agents (like halothane) this may be achieved only via direct effects of putative antagonists at the carotid body, as discussed earlier, with background arousal levels being less important.

## CONCLUSIONS

It is unknown why K2P channels should play such a ubiquitous role in so many aspects of respiratory function relevant to clinical anesthesia. This includes their roles in oxygen sensing in the carotid body, carbon dioxide sensing in the central chemoreceptor and central respiratory rhythm generation, regulation of the pulmonary vasculature, upper airway tone, and sleep-wake cycling. Indeed, the extent of K2P channel expression raises the interesting question as to why they do not seem to be involved in the other organ key organ involved in oxygen sensing, namely the kidney. A recent review of hypoxia inducible factor (HIF) observed no links between this system and K2P channels,^118^ and laboratory investigations do not link HIF manipulation in the kidney to HVR.^119^ Nevertheless, K2P channels are expressed in the kidney and it is possible they mediate other as yet unexplored functions.

All this presents numerous therapeutic targets to: prevent anesthetic depression of hypoxic and hypercapnic chemoreflexes, maintain ventilatory drive during and after surgery, optimize ventilation-perfusion matching, reverse the anesthetic exacerbation of airway obstruction in sleep apnea, and potentially reverse hypnotic effects of anesthesia postsurgery. Actual drug development is in its infancy, but any therapies will need to be very specific for the relevant K2P subtypes, given their ubiquity across the systems, so as to avoid deleterious side effects. A particular, arguably unique property of K2P (TASK) channels, discovered first in the carotid body, is their ability to respond to both hypoxia and anesthetics. This makes them especially important as explanations for whole-body observations of anesthetic-respiratory interactions, as well as clinically relevant targets for therapeutic manipulation.

## DISCLOSURES

**Conflicts of Interest:** J. J. Pandit is Editor-in-Chief of *Anesthesia & Analgesia*. **Funding:** None. **This manuscript was handled by:** Christina M. Pabelick, MD.

## Supplementary Material
