# Supplementary material for: The “TASK” of Breathing: Anesthetic Relevance of Background Two-Pore Domain Potassium Channels as Therapeutic Targets for Respiratory Control
Source: Anesth Analg. 2025 Feb 13;140(6):1414–25. doi: 10.1213/ANE.0000000000007365 (PMC12063681; doi:10.1213/ANE.0000000000007365)
Supplement: Supplementary file 1 [file ane-140-1414-s001.pdf]

SUPPLEMENTARY DIGITAL CONTENT

ONLINE SUPPLEMENT S1. Dendrogram of human two-pore domain (K2P) channels

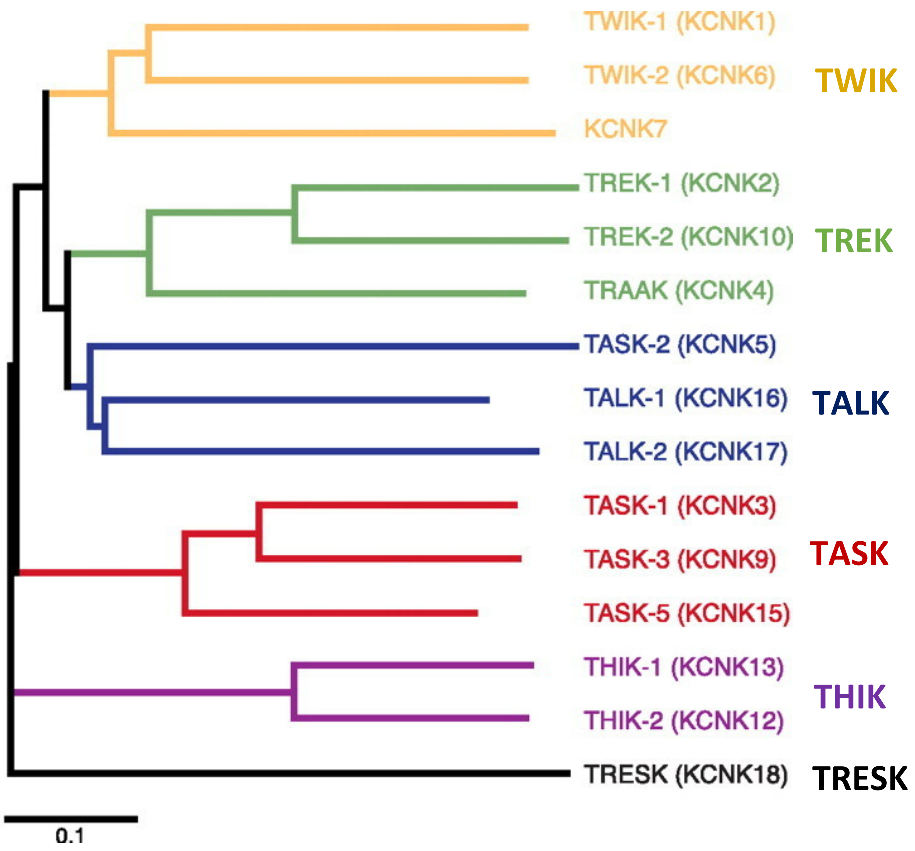

ONLINE SUPPLEMENT S1. Dendrogram of human two-pore domain (K2P) channels, reproduced from Enyedi and Czirjak[1] and edited to include subfamily titles. The six subfamilies, indicated in different colors, were categorized based on sequence and functional variation; sequence variation between subfamilies is substantial despite their sharing the same basic molecular structures[1]. The conventional and systematic (HUGO) names are both indicated for each channel.

ONLINE SUPPLEMENT S2. Summary of some anesthetic interaction at K2P channels

Table S1: Volatile Anesthetics

**Anesthetic**	**K2P channel subtype/s**	**Species**	**Activity**	**Concentrations Used**	**EC50 /IC50**	**Recording configuration**	**Cell type**	**Source**
Halothane	Native TASK<br><br>i.e. mostly TASK-1/3 heterodimer; some homodimers	rat	Activation (strong)	0 -1.2mM		whole cell patch clamp	rat carotid glomus cell	Pandit et al., 2020[2]
Isoflurane			Activation (less than halothane)	0 – 1.4mM				
Halothane + Isoflurane			Activation (intermediate)	0.36mM + 0.42mM; 0.6mM% + 0.7mM 0.96mM+1.12mM				
Halothane	TASK-1		Activation	0 – 1.2mM			HEK-293	
Isoflurane			Activation (less than halothane)	0 – 1.2mM				
Halothane + Isoflurane			Activation (intermediate)	1.2mM halothane with 0.42–1.4mM% isoflurane				
Halothane	TASK-1	human; rat	Activation	1 mM		two electrode voltage clamp	*Xenopus* oocyte	Putzke et al., 2007[3]
Sevoflurane		human						
Isoflurane; its purified enantiomers								
Halothane	TASK-1	human	Activation	0.05 - 0.53 mM	0.13 ± 0.04 mM	whole cell patch clamp	HEK-293	Andres-Enguix et al., 2007[4]
Chloroform			Inhibition at supraclinical concentrations	0.66 - 6.6 mM	3.2 ± 1.8 mM			
Isoflurane	TASK-1/3 heterodimer	rat	Activation (106 ± 7%)	0.8 mM		whole cell patch clamp	HEK-293	Berg et al., 2004[5]
	TASK-1		Inhibition (-15 ± 2%)	0.8 mM				
Halothane	TASK-1	rat	Activation	0.3 mM		whole cell patch clamp	HEK-293 + TRH-R1 receptor	Talley and Bayliss, 2002[6]
Halothane	TASK-1	human	Activation	1 mM		whole cell patch clamp	COS	Patel et al., 1999[7]
Isoflurane			Activation	2 mM				
Chloroform			Minimal inhibition	0.8 mM				
Diethyl ether			Inhibition	0.5 mM				
Halothane	TASK-3	rat	Activation	0.2 - 2.7 mM		Ussing chamber voltage clamp	Fischer rat thyroid cell monolayer	Luethy et al., 2017[8]
Isoflurane				0.2 - 2.7 mM	363 µM			
Desflurane				0.2 - 2.2 mM	433 µM			
Sevoflurane				0.1 - 1.1 mM	265 µM			
Chloral hydrate				0.1 – 25.9 mM	7141 µM			
Chloroform	TASK-3	human	Activation		0.5 ± 0.2 mM	whole cell patch clamp	HEK-293	

Halothane					0.4 ± 0.4 mM			Andres-Enguix et al., 2007[4]
Isoflurane	TASK-3	rat	Activation (138 ± 25%)	0.8 mM		whole cell patch clamp	HEK-293	Berg et al., 2004[5]
Halothane	TASK-3	human	Activation (65.6 ±15.2%)	1 mM		two-electrode voltage clamp	*Xenopus* oocyte	Meadows & Randall, 2001[9]
Halothane	TASK-3	rat	Activation	0.3 mM		whole cell patch clamp	HEK-293 + TRH-R1 receptor	Talley and Bayliss, 2002[6]
Isoflurane	TASK-3	human	Activation (4.4 ± 1.5%)	769 µM		whole cell patch clamp	*Xenopus* oocyte	Liu et al., 2004[10]
Halothane	TASK-2	human	Activation	50 - 1000 µM		whole cell patch clamp	*Xenopus* oocyte	Gray et al., 2000[11]
Isoflurane				500 - 1000 µM				
Desflurane				1160 µM				
Enflurane				980 µM				
Chloroform				1400 µM				
Chloroform	TREK-1	human	Activation	0.3 mM; 1 mM		whole cell patch clamp	COS	Patel et al., 1999[9]
				0.8 mM		outside-out patch clamp		
		mouse		0.8 mM		whole cell patch clamp		
Halothane		human		0.1 - 1 mM		whole cell patch clamp		
				0.2 - 1 mM		outside-out patch clamp		
				1 mM		inside-out patch clamp		
		mouse		1 mM		whole cell patch clamp		
Isoflurane		human		0.3 mM; 1 mM		whole cell patch clamp		
		mouse		2 mM				
Diethyl ether		mouse		0.6 mM		whole cell patch clamp		
Halothane	TREK-1	human	Activation (35 ± 3%)	0.144mM		whole cell patch clamp	tsA201	Gruss et al., 2004[12]
			Activation (25 ± 3%)	0.144mM		outside-out patch clamp		
Halothane	TREK-like current	mouse	Activation	2 mM		outside-out patch clamp	striatal neuron	Heurteaux et al., 2004[13]
Chloral hydrate	TREK-1	human	Transient activation then rapid inhibition	10 – 40 mM		whole cell patch clamp	CHO	Harinath and Sikdar, 2004[14]
Chloroform	TREK-2	human	Activation (1.8 ± 0.1fold)	1 mM		whole cell patch clamp	COS	Lesage et al., 2000[15]
Halothane			Activation (2.3 ± 0.3fold)					
Isoflurane			Activation (1.91 ± 0.1fold)					

Chloral hydrate	TRAAK	human	Activation	30 mM		whole cell patch clamp	CHO	Harinath and Sikdar, 2004[14]
Chloroform	TRAAK	mouse	no effect	0.8 mM		whole cell patch clamp	COS	Patel et al., 1999[7]
Halothane				1 mM				
Isoflurane				2 mM				
Diethyl ether				0.6 mM				
Halothane	TRESK	human	Activation		300 ± 29 µM	two-electrode voltage clamp	*Xenopus* oocyte	Liu et al., 2004[10]
				385 µM		single channel patch clamp	COS-7	
Isoflurane					162 ± 32 µM	two-electrode voltage clamp	*Xenopus* oocyte	
				450 µM		single channel patch clamp	COS-7	
Sevoflurane					224 ± 35 µM	two-electrode voltage clamp	*Xenopus* oocyte	
Desflurane					658 ± 138 µM			
Isoflurane; enantiomers	TRESK	mouse	Activation		226 µM (isoflurane)	whole cell patch clamp	*Xenopus* oocyte	Keshavaprasad et al., 2005[16]
		rat			346 µM (isoflurane)			
Halothane	THIK-1	rat	Inhibition	5 mM	2.83 mM	two-electrode voltage clamp	*Xenopus* oocyte	Rajan et al., 2001[17]

Table S2:

Insoluble Anesthetic gases

Anesthetic	K2P channel subtype/s	Species	Activity	Concentrations applied	EC50 /IC50	Method	Cell type	Source
Xenon	TASK-3	human	No effect	80%		whole cell patch clamp	tsA201	Gruss et al., 2004[12]
Cyclopropane				10%; 80%				
Nitrous oxide				80%				
Xenon	TREK-1		Activation (35 ± 2%)	80%		whole cell patch clamp		
			Activation (27 ± 2%)			outside-out patch clamp		
Cyclopropane			Activation (35 ± 5%)	10%		whole cell patch clamp		
Nitrous oxide			Activation (28 ± 2%)	80%				
Nitrous oxide	TRESK	human	Activation (13.6 ± 4.5%)	70%		two-electrode voltage clamp	*Xenopus* oocytes	Liu et al., 2004[10]

Table S3: Intravenous Anesthetics

Anesthetic	K2P channel subtype/s	Species	Activity	Concentrations Used	EC50 /IC50	Method	Cell type	Source
Etomidate	TASK-1	human	Inhibition at supraclinical concentrations	50 - 200 µM	119uM	whole cell patch clamp	*Xenopus* oocyte	Putzke et al., 2007[3]
Propofol			No effect	50 µM; 200 µM				
Propofol	Native TASK<br>i.e. mostly TASK-1/TASK-3 heterodimer; some homodimers)	rat	No effect	100 µM		cell-attached patch clamp	rat type 1 glomus cell	O'Donohoe et al., 2019[18]
Etomidate	TASK-3	human	Inhibition at supraclinical concentrations	0 – 200 µM	128 µM	whole cell patch clamp	*Xenopus* oocyte	Putzke et al., 2007[3]
Propofol			No effect	50 µM; 200 µM				
Pentobarbital	TASK-3	human	Inhibition (-4.3 ± 2.6%)	100 µM (supraclinical)		two-electrode voltage clamp	*Xenopus* oocyte	Meadows & Randall, 2001[9]
Ketamine			Inhibition (-7.3 ± 2.2%)					
Alphaxolone			Inhibition (-49.2 ± 6.2%)					
Alpha-chloralose	TASK-3	rat	Inhibition	Not indicated		Ussing chamber voltage clamp	Fischer rat thyroid cell monolayers	Luethy et al., 2017[8]
Etomidate	TRESK	human	Inhibition (-30.5 ±4.8%)	100 µM (supraclinical)		two-electrode voltage clamp	*Xenopus* oocyte	Liu et al., 2004[10]
Pentobarbital			Inhibition (-10.4 ±4.3%)					
Ketamine			Inhibition (-14.5 ±2.7%)					
Alphaxolone			Inhibition (-45.4 ±2.3%)					

Table S4: Other notable drugs

Substance	Channel	Species	Activity	Concentrations Used	EC50/ IC50	Method	Cell type	Source
Doxapram	TASK-1/3 heterodimer	rat	Inhibition		9 µM	two-electrode voltage clamp	*Xenopus* oocyte	Cotten et al., 2006[19]
	TASK-1				410 nM			
	TASK-3				37 µM			
Gabapentin	TRESK	human	Inhibition (4.2 ± 0.4%)	100 µM		two-electrode voltage clamp	*Xenopus* oocyte	Liu et al., 2004[10]

Online Supplement S3. Summary of knockout (K/O) mice work on anesthetic sensitivity

**Channel**	**Effects of KO on sensitivity to anesthetics and/or ventilation**	**Source**
**TREK-1**	Increased latency to loss of righting reflex (LORR) and minimum alveolar concentration (MAC) with chloroform, halothane, sevoflurane, desflurane; no change with pentobarbital.	Heurteaux et al., 2004[13]
**TREK-1**<br>**TREK-2**<br>**TREK-1; TREK-2** (double KO)	No effects on induction or emergence values in responses to tail clamp, and no change to MAC values, for isoflurane and halothane	Spencer et al., 2023[20]
**TRESK**	Increase in MAC with isoflurane; no significant difference in MAC under halothane, sevoflurane or desflurane.	Chae et al., 2010[21]
**TASK-1**	Increased MAC under both halothane and isoflurane.	Linden et al., 2006[22]
**TASK-1**	Prolonged LORR to propofol and pentobarbital.	Linden et al., 2008[23]
**TASK-3**	Increased MAC with halothane; no significant change in response to isoflurane, propofol or dexmedetomidine.	Linden et al., 2007[24]
**TASK-3**	Substantial increase in MAC with halothane.	Pang et al., 2009[25]
**TASK-1**<br>**TASK-3**<br>**TASK-1; TASK-3**	In all KO lines, increased MAC with both halothane and isoflurane; increased latency to LORR with only halothane. No difference in results between KO lines.<br><br>'In motoneurons from all knock-out mice lines, TASK-like currents were reduced and cells were less sensitive to hyperpolarizing effects of halothane and isoflurane.'	Lazarenko et al., 2010[26]
**TASK-1**<br>**TASK-3**<br>**TASK-1; TASK-3**	Whole animal plethysmography revealed 'hypersensitivity to low CO_2 and reduced ventilatory response to higher CO_2, such that the overall [hypercapnic ventilatory] reflex was blunted'. There were no differences in increases in ventilation under hyperoxic hypercapnia (hyperoxia was used to isolate the central chemoreceptor effect).<br><br>In a working heart-brainstem preparation, there was reduced sensitivity to hypocapnic alkalosis, as measured by phrenic nerve activity under different perfusate CO_2 levels.	Bayliss et al., 2015[27]

	These trends were the same for the three KO lines.	
**TASK-2**	Whole animal plethysmography revealed hypersensitivity of ventilatory rate to low CO_2 and loss of long-term hypoxia-induced respiratory decrease in the KO mice. The acute carotid body-mediated hypoxia-induced increase in ventilatory rate was maintained.	Gestreau et al., 2010[28]
**TASK-2**	No effect on MAC with desflurane, halothane, isoflurane.	Gerstin et al., 2003[29]
**KCNK7**	No effect on MAC with isoflurane, sevoflurane, desflurane	Yost et al., 2008[30]

Online Supplement S4. Supplementary references

S1. Enyedi P, Czirják G. Molecular background of leak K^+ currents: two-pore domain potassium channels. *Physiol Rev*. 2021; 90: 559-605.

S2. Pandit JJ, Huskens N, O'Donohoe PB, Turner PJ, Buckler KJ. Competitive interactions between halothane and isoflurane at the carotid body and TASK channels. *Anesthesiology*. 2020; 133: 1046-59.

S3. Putzke C¸ Hanley PJ, Schlichthörl G, et al.. Differential effects of volatile and intravenous anaesthetics on the activity of human TASK-1. *Am J Physiol Cell Physiol.* 2007; 293(4): C1319-C1326.

S4. Andres-Enguix I, Caley A, Yustos R, et al. Determinants of the anesthetic sensitivity of two-pore domain acid-sensitive potassium channels: molecular cloning of an anesthetic-activated potassium channel from Lymnaea stagnalis. *J Biol Chem*. 2007; 282(29): 20977-20990.

S5. Berg AP, Talley EM, Manger JP, Bayliss DA. Motoneurons express heteromeric TWIK-related acid-sensitive K+ (TASK) channels containing TASK-1 (KCNK3) and TASK-3 (KCNK9) subunits. *J Neurosci.* 2004; 24: 6693-6702.

S6. Talley EM, Bayliss DA. Modulation of TASK-1 (Kcnk3) and TASK-3 (Kcnk9) potassium channels: volatile anesthetics and neurotransmitters share a molecular site of action. *J Biol Chem*. 2002; 277(20):17733-17742.

S7. Patel AJ, Honoré E, Lesage F, Fink M, Romey G, Lazdunski M. Inhalational anesthetics activate two-pore-domain background K^+ channels. *Nat. Neurosci.* 1999; 2(5): 422-426.

S8. Luethy A, Boghosian JD, Srikantha R, Cotten JF. Halogenated ether, alcohol, and alkane anesthetics activate TASK-3 tandem pore potassium channels likely through a common mechanism. *Mol Pharmacol.* 2017; 91(6): 620-629.

S9. Meadows HJ, Randall AD. Functional characterisation of human TASK-3, an acid-sensitive two-pore domain potassium channel. *Neuropharmacology.* 2001; 40(4): 551-559.

S10. Liu C, Au J, Liao H, Cotten J, Yost CS. Potent activation of the human tandem pore domain K channel TRESK with clinical concentrations of volatile anesthetics. *Anesth Analg.* 2004; 99(6): 1715-1722.

S11. Gray AT, Zhao BB, Kindler CH, et al. Volatile anesthetics activate the human tandem pore domain baseline K^+ channel KCNK5. *Anesthesiology.* 2000; 92(6): 1722-1730.

S12. Gruss M, Bushell TJ, Bright DP, Lieb WR, Mathie A, Franks NP. Two-pore-domain K^+ channels are a novel target for the anesthetic gases xenon, nitrous oxide, and cyclopropane. *Mol Pharmacol.* 2004; 65(2): 443-452.

S13. Heurteaux C, Guy N, Laigle C, et al. TREK-1, a K+ channel involved in neuroprotection and general anesthesia. *EMBO J.* 2004; 23(13): 2684-2695.

S14. Harinath S, Sikdar SK. Trichloroethanol enhances the activity of recombinant human TREK-1 and TRAAK channels. *Neuropharmacology.* 2004; 46(5): 750-760.

S15. Lesage F, Terrenoire C, Romey G, Lazdunski M. Human TREK2, a 2P domain mechano-sensitive K+ channel with multiple regulations by polyunsaturated fatty acids, lysophospholipids, and Gs, Gi, and Gq protein-coupled receptors. *J Biol Chem.* 2000;.275(37): 28398-28405.

S16. Keshavaprasad B, Liu C, Au JD, Kindler CH, Cotten JF, Yost CS. Species-specific differences in response to anesthetics and other modulators by the K2P channel TRESK. *Anesth Analg.* 2005; 101(4): 1042-1049.

S17. Rajan S, Wischmeyer E, Karschin C, et al. THIK-1 and THIK-2, a novel subfamily of tandem pore domain K+ Channels. *J Biol Chem*. 2001; 276(10): 7302-7311.

S18. O'Donohoe PB, Turner PJ, Huskens N, Buckler KJ, Pandit JJ. Influence of propofol on isolated neonatal rat carotid body glomus cell response to hypoxia and hypercapnia. *Respir Physiol Neurobiol.* 2019; 260: 17-27.

S19. Cotten JF, Keshavaprasad B, Laster MJ, Eger EI 2nd, Yost CS. The ventilatory stimulant doxapram inhibits TASK tandem pore (K2P) potassium channel function but does not affect minimum alveolar anesthetic concentration. *Anesth Analg.* 2006; 102(3): 779-785.

S20. Spencer KA, Woods CB, Worstman HM, et al. TREK-1 and TREK-2 knockout mice are not resistant to halothane or isoflurane. *Anesthesiology*. 2023; 139(1): 63–76.

S21. Chae, Y.J. *et al.* Discrete change in volatile anesthetic sensitivity in mice with inactivated tandem pore potassium ion channel TRESK. *Anesthesiology* 2010; **113**: 1326-1337.

S22. Linden AM, Aller MI, Leppä E, et al. The in vivo contributions of TASK-1-containing channels to the actions of inhalation anesthetics, the alpha (2) adrenergic sedative dexmedetomidine, and cannabinoid agonists. *J Pharmacol Exp Ther*. 2006; 317(2): 615-626.

S23. Linden AM, Aller MI, Leppä E, Rosenberg PH, Wisden W, Korpi ER. K+ channel TASK-1 knockout mice show enhanced sensitivities to ataxic and hypnotic effects of GABA(A) receptor ligands. *J Pharmacol Exp Ther*. 2008; 327(1): 277-286.

S25. Linden AM, Sandu C, Aller MI, et al. TASK-3 knockout mice exhibit exaggerated nocturnal activity, impairments in cognitive functions, and reduced sensitivity to inhalation anesthetics. *J Pharmacol Exp Ther.* 2007; 323(3): 924-934.

S26. Pang DS, Robledo CJ, Carr DR, et al. An unexpected role for TASK-3 potassium channels in network oscillations with implications for sleep mechanisms and anesthetic action. *Proc Natl Acad Sci USA*. 2009; 106(41): 17546-17551.

S27. Lazarenko RM, Willcox SC, Shu S, et al. Motoneuronal TASK channels contribute to immobilizing effects of inhalational general anesthetics. *J Neurosci*. 2010;30(22):7691-7704.

S28. Bayliss DA, Barhanin J, Gestreau C, Guyenet PG. The role of pH-sensitive TASK channels in central respiratory chemoreception. *Pflugers Arch.* 2015; 467: 917-929.

S29. Gestreau C, Heitzmann D, Thomas J, et al. Task2 potassium channels set central respiratory CO2 and O2 sensitivity. *Proc Natl Acad Sci USA.* 2010; 107(5): 2325-2330.

S30. Gerstin KM, Gong DH, Abdallah M, et. al. Mutation of KCNK5 or Kir3.2 potassium channels in mice does not change minimum alveolar anesthetic concentration. *Anesth Analg.* 2003; 96(5): 1345-1349.

S31. Yost CS, Oh I, Eger EI 2nd, Sonner JM. Knockout of the gene encoding the K(2P) channel KCNK7 does not alter volatile anesthetic sensitivity. *Behav Brain Res.* 2008; 193(2): 192-196.
